# Supplementary material for: Are carbon emissions trading and green financial instruments synergistic? -Comprehensive quantitative research based on content analysis
Source: PLoS One. 2024 Mar 7;19(3):e0298601. doi: 10.1371/journal.pone.0298601 (PMC10919601; doi:10.1371/journal.pone.0298601)
Supplement: S1 Data — (ZIP) [file pone.0298601.s001.zip › Data/地级市Z_F工作报告.zip!/╒π╜¡/─■▓¿╩╨/└┤╫╘╣1⁄2╓┌║┼í╛┬φ┐╦╩2╛▌═°í┐_2014.docx]

各位代表：

　　现在，我代表宁波市人民政府向大会报告工作，请予审议，并请市政协委员和其他列席人员提出意见。

　　一、2013年工作回顾

　　一年来，面对国内外错综复杂的经济形势、频繁的自然灾害特别是洪涝灾害的严重影响，市政府在中共宁波市委的领导下，全面贯彻落实党的十八大和十八届二中、三中全会精神，深入实施“六个加快”战略，按照“稳中求进、进中求好”的工作总基调，全力以赴稳增长、促转型、惠民生，千方百计扩总量、促增量、提质量，与全市人民一道，齐心协力、攻坚克难，各项工作取得了扎实成效，较好地完成了市十四届人大三次会议确定的目标任务。初步测算，实现地区生产总值7100亿元，增长8%以上；完成地方财政收入792.4亿元，增长9.2%；市区居民人均可支配收入达到41700元，农村居民人均纯收入达到20500元，分别增长10%以上和11%左右；居民消费价格指数102.2%。民生实事全面完成。

　　一年来，我们主要做了六个方面的工作：

　　（一）保持经济稳定增长。紧紧围绕扩规模、优结构、提效益，狠抓有效投资。完成固定资产投资3423亿元、增长18%，其中工业投资增长30.3%、民间投资比重超过50%；引进十亿元以上投资项目31个，其中百亿元项目3个。钓鱼台·美高梅“中国假期”、华强·中华复兴文化园等项目签约，华侨城文化旅游等103个重点工程项目开工建设，海越新材料、中烟宁波易地技改等项目进展顺利，上海大众汽车一期、南车超级电容器等项目建成投产。针对消费市场的新形势新业态，综合施策，完善城乡现代商业服务体系，发展电子商务，推进市镇商贸中心、商业特色街和商业示范社区建设，完成社会消费品零售总额2630亿元、增长13%。面对严峻的外贸环境，做好产品和市场的优化提升文章，开展市场采购监管模式和跨境贸易电子商务服务试点，实施“外贸实力效益工程”和“外贸育苗工程”，加快国家进口贸易促进创新示范区建设，外贸自营进出口总额突破1000亿美元，出口预计增长6%以上。

　　（二）加快转型升级步伐。强化创新驱动发展，启动新材料科技城和国家高新区“一区多园”建设，实施“科技领航计划”、“智团创业计划”，宁波国家级文化和科技融合示范基地获批，复旦大学宁波研究院、海洋电子研究院、西安电子科技大学宁波信息技术研究院启动建设，石墨烯产业化应用开发、碳纤维规模化生产等项目落地转化，发明专利授权量增长10%，研究与试验发展经费支出占地区生产总值比重提高到2.2%。推进海外高层次人才引进“3315计划”和“百校千企”人才合作培养，出台新材料科技城人才管理改革试验区政策，“千人计划”余姚产业园和宁波人力资源服务产业园成为省级产业园，新增人才18万人。着力建设工业强市，实施“四换三名三创”工程，推进战略性新兴产业发展，开展自动化（智能化）成套装备改造试点，推动“个转企、小升规、规改股、股上市”，战略性新兴产业、高新技术产业产值占规上工业总产值比重分别达到25.5%和30%，新产品产值率达到22%，关停落后产能企业199家。扎实推进质量强市建设，成为首批全国质量强市示范城市。加快建筑业转型发展，建筑业总产值突破3000亿元。

　　现代物流、文化创意、金融、旅游等产业加快发展，宁波广告产业园区成为国家广告产业试点园区，金融机构本外币存贷款余额分别增长10.9%和11.5%，服务业增加值占地区生产总值比重提高到43.5%。新增标准化粮食生产功能区10万亩、现代农业园区60个，建成高标准基本农田35万亩，家庭农场成为全国五大发展“范本”之一。加快智慧城市建设，智慧物流、智慧健康、智慧社会管理试点成效明显。浙江海洋经济发展核心示范区建设稳步推进，海洋经济科技支撑体系基本形成，海洋新兴产业加速发展，海洋资源产权交易改革积极推进。港口货物和集装箱吞吐量分别增长9.5%和7%。

　　（三）推进城乡协调发展。启动城市总体规划修改和报批工作，着力以规划引领城乡统筹发展、区域布局优化、发展空间拓展和城市功能提升。深入实施现代都市“50100工程”，东部新城、南部新城、镇海新城、北仑滨海新城、东钱湖旅游度假区等区块建设稳步推进。加强中心城区改造提升，开展交通拥堵、户外广告、城市主干道和背街小巷综合整治，“三江六岸”滨江休闲带工程启动段建成投用，打通“断头路”三年行动计划顺利完成。强力推进“三改一拆”，拆改面积大幅超额完成年度目标。都市圈南北两翼加快发展，余姚高铁新城、慈溪文化商务区、奉化滨海新区、浙台（象山石浦）经贸合作区建设步伐加快，宁波三门湾区域发展规划出台实施。加快幸福美丽新家园建设，“三村一线”创建、农房“两改”、“双清”等工作有效推进，新启动农村集中住房建设项目76个、省级农房改造建设示范村创建项目22个，农村集体土地所有权登记发证全面完成。加大低收入农户帮扶力度，相对欠发达地区生产生活条件持续改善。交通网络建设成效明显，杭甬客专、铁路宁波站、穿山疏港高速、大榭第二大桥建成投用，轨道交通1号线和2号线一期、南北环快速路等项目有序推进，杭州湾跨海大桥杭甬高速连接线开工建设，成为国家公交都市创建示范城市。

　　（四）激发改革开放活力。推进资源要素配置市场化改革，强化产业项目用地准入管理，排污权有偿使用交易工作有序开展。大力发展“天使投资”，推动金融机构改革重组，民间融资规范化、阳光化试点稳步实施。深化服务业综合改革试点，积极搭建商品交易平台。农村集体林权制度、集体经济股份合作制改革继续深化。推进卫星城市改革发展，中心镇的管理权限和行政执法权限进一步扩大。加强对外开放平台建设，推进重点开发区域整合提升，国际卫生港创建进展顺利，口岸功能进一步增强。宁波进口商品展示交易中心投入使用，梅山保税港区汽车整车进口口岸通过验收。加强国际国内招商力量，推进招商选资，实际利用外资32.7亿美元，引进内资、浙商甬商回归投资项目实到资金超额完成年度目标。积极推动企业“走出去”开展境外收购、生产制造和贸易基地建设，新批境外企业和机构206家，境外中方投资额增长20%。离岸服务外包执行额增长47%。国内区域合作继续深化，援疆、援藏、援黔及山海协作等扎实推进。

　　（五）改善社会民生福祉。把生态环境建设放在突出位置，实施“三治理一提高”行动，开展国家低碳城市试点，强化森林资源保护，推进“四边三化”行动和建筑渣土、建设工程扬尘整治。淘汰高污染燃料使用设施409台，关停重污染企业936家，“禁燃区”建设三年行动计划和主要污染物减排目标顺利完成，宁海成为国家级生态县。开展“文明宁波”系列活动，公民思想道德建设不断加强，建成81890志愿服务中心。推进公共文化服务体系示范区建设，县（市）区全部成为省级文化先进县，建成示范性农村文化礼堂150家，宁波文化广场建成投用，文化精品创作、文化遗产保护取得新成果。哲学社会科学、档案史志等事业取得新进展。加快推进教育现代化，开展教育国际合作与交流综合改革试验区建设，落实高教协同创新计划，深化招生评价制度和民办教育综合改革，构建现代服务业公共职业培训平台，职业教育“校企通”平台功能更加完善。竞技体育水平稳步提升，宁波籍运动员在第十二届全运会上取得历史最好成绩。推进国家级创业型城市和充分就业县（市）区建设，扶持高校毕业生就业创业，职业技能培训21.5万人次，城镇新增就业16.1万人，新增创业实体10.4万家，城镇登记失业率2.16%，创历史新低。市级公立医院综合改革全面启动，县级公立医院综合改革扎实推进，新十大医疗卫生基础设施加快建设，通过国家卫生城市复查。城乡居民医疗保险统筹实质性启动。本地户籍人口养老保障参保率提高到87.2%，职工医保、居民医保覆盖面均达到95%以上，新农合覆盖面达到98.3%。企业退休人员养老金、城乡居民基础养老金待遇和职工医保、居民医保、新农合住院报销比例继续提高，困难群众基本生活保障水平逐步提升，计划生育特殊家庭扶助制度进一步完善。实现省级扶残助残爱心城市创建全覆盖。新开工保障性安居工程140.7万平方米、1.5万套。创新基层公共治理形式，协商民主和法制建设深入开展。深化重大决策社会稳定风险评估，畅通群众利益诉求表达和回应渠道，推行多元化调解模式，流动人口管理服务工作得到加强。深化“平安宁波”建设，推进社会治安重点地区整治和立体化治安防控体系建设，严厉打击各类违法犯罪活动。全面深化安全生产大排查大整治，安全生产形势保持稳定。食品药品监管力度加大。支持国防建设，国防动员、双拥工作进一步深化。支持工会、共青团、妇联、慈善、红十字、关工委等群团组织发挥作用，人口计生、老龄、民族、宗教、侨务、外事、港澳台等工作得到加强。

　　（六）提高政府服务效能。扎实开展党的群众路线教育实践活动，全面落实中央“八项规定”、省委“28条办法”和市委“20条措施”，认真查摆“四风”方面突出问题，深入开展“正风肃纪”专项行动，大力整治庸懒散奢等不良风气。精简会议活动和文件简报，实施市长电话“一号通”工程，推进政务公开、信息公开和新闻发布，政府透明度位居省会城市、计划单列市首位。严格清理楼堂馆所和办公用房，大力压缩一般性支出，“三公”经费大幅下降。主动向市人大报告工作，向市政协通报重大事项，认真接受人大、政协和社会各方面的监督，办理代表建议560件、政协提案588件，提交地方性法规议案3件。制定修改政府规章6件，办理行政复议案件263件。加强行政规范性文件和行政机关合同管理，规范行政机关执法权限，推进城市管理相对集中行政处罚权工作，北仑区率先实施城乡一体化城市管理综合行政执法体制。推进行政审批标准化建设，规范工程建设领域中介服务，统一下放行政审批事项263项，市行政服务中心窗口受理事项比例达到100%、办理事项比例达到93.7%。开展“十二五”规划纲要实施情况中期评估，发展战略谋划和规划编制工作得到加强。加强审计监督和行政监察，廉政风险防控机制进一步完善。

　　各位代表！去年我市遭遇了禽流感、高温干旱、台风、洪涝等灾害，特别是“菲特”强台风带来的洪涝灾害，影响之广、损失之重历史罕见。在特大洪涝灾害面前，我们把抗洪救灾作为压倒一切的头等大事，把保障群众生命安全放在第一位，迅速启动防台防汛应急响应，紧急组织抢险力量和救灾物资，第一时间投入灾区抗洪救灾，尽快恢复灾区生活秩序和重要基础设施运行。洪灾过后，出台“1+11”扶持政策和“一对一”帮扶余姚重建措施，复产重建工作有效、有序、有力推进。在抗洪救灾中，全市上下团结一心、众志成城，基层干部群众奋战一线，驻浙部队、武警官兵和公安干警冲锋陷阵，广大志愿者无私奉献，社会各界慷慨捐助，各行各业全力支持，展现了“一方有难、八方支援”的良好风貌，为打赢抗洪救灾这场硬仗作出了不懈努力。

　　各位代表！在严峻复杂的外部环境和严重的自然灾害影响下，去年我市取得上述成绩来之不易。这是党中央国务院、省委省政府和市委正确领导的结果，是全市人民共同努力的结果。在此，我代表市人民政府，向全市人民，向市人大代表、市政协委员，向离退休老干部，向各民主党派、工商联和社会各界人士，向驻甬人民解放军和武警部队官兵，向“宁波帮”和帮宁波人士，以及所有关心支持宁波发展的海内外朋友们，表示衷心的感谢!

　　同时，我们也清醒地看到，我市发展中还存在不少问题，政府工作还有许多不足。去年，地区生产总值、进口和节能指标没有完成预期目标。创新驱动基础不够坚实，战略性新兴产业和现代服务业发展亟待加快；中心城区集聚辐射带动能力和建设品质不够高，城乡基础设施尤其是防洪排涝设施建设滞后；重点领域和关键环节改革还不到位，对外开放的新优势远未建立；生态环境、教育医疗、交通出行、养老服务、食品质量、安全生产等方面还存在不少问题；政府职能转变、社会治理创新、应急救灾能力都有待加强，一些政府工作人员的服务意识、服务能力不强，改进工作作风、密切联系群众的长效机制需要进一步完善。对此，我们将针对性地采取有力措施，认真加以改进。

　　二、全力实施经济社会转型发展三年行动计划

　　今后三年，是我市实现“两个基本”、建设“四好示范区”的关键时期。为深入实施“六个加快”战略，认真落实“双驱动四治理”决策部署，我们结合“十二五”规划纲要实施情况中期评估结果和当前发展的新形势、新要求，研究制定了经济社会转型发展三年行动计划，以重大项目为抓手，大力推进“五水共治”、现代产业培育、城市功能提升、民生福祉改善和发展基础强化“五项工程”建设，为实现市第十二次党代会提出的目标任务奠定坚实基础。主要目标和任务包括：

　　（一）大力实施“五水共治”工程。一是治污水。重点整治黑河、臭河、垃圾河，加快城乡污水、垃圾处理设施建设，推进高污染行业整治提升和农业面源污染治理。二是防洪水。以江河堤防加固、骨干河道整治和水闸、泵站配套为重点，加快“治水强基”工程建设。三是排涝水。推进水库除险加固，打通“断头河”，开辟新河道，清理疏浚河床，加快应急强排设施建设。四是保供水。加强饮用水源地生态保护，推进区域饮水工程和供水管网建设。五是抓节水。着力抓好减少漏损、再生利用、改装器具等工作，合理利用水资源。

　　到2016年，以“五水共治”倒逼转型发展的工作机制更加健全，水环境质量、防洪排涝能力大幅提升，供水节水体系更加完善。水环境功能区水质达标率达到71%，力争城镇截污纳管基本覆盖，农村污水处理、生活垃圾集中处理基本覆盖；防洪减灾基础设施滞后局面切实改变，“三江”干流堤防全线封闭；洪涝调蓄能力大大增强，初步形成流域和主要平原的主干排涝体系。

　　（二）大力实施现代产业培育工程。一是加快现代服务业跨越式发展。重点培育国际贸易、现代物流、现代金融、现代会展、文化创意、旅游休闲、健康养老等产业，大力发展总部经济、电子商务、服务外包等新兴业态。二是推进工业强市建设。做强做大战略性新兴产业，全面提升汽车及零部件、高端装备等优势制造业，整合优化石化等产业，培育大企业大集团。三是积极发展现代农业。推进粮食生产功能区、现代农业园区和海洋渔业综合基地建设，做优现代都市农业、休闲农业和精品农业。

　　到2016年，基本形成以城市经济为主体、现代服务业为引领、先进制造业为支撑、现代农业为基础，三次产业融合协调发展的现代产业体系。服务业增加值占地区生产总值比重达到46%，形成一批战略性新兴产业龙头企业和产业集聚区，海洋经济生产总值达到2700亿元，现代农业发展水平全面提高。

　　（三）大力实施城市功能提升工程。一是推进功能区块开发建设。全面建成东部新城核心区、南部商务区二期，加快湾头区块、鄞奉片区、南站综合客运枢纽周边区块建设，启动东部新城东片区、姚江新区、甬江两岸、城西等区块开发，推进历史文化街区保护提升、老街区改造和老旧商务楼宇品质提升。二是促进高端要素集聚。加快新材料科技城、国家高新区“一区多园”、电子商务“一城两区一中心”、国际贸易展览中心拓展区、进口商品展示交易中心、宁南贸易物流区等建设，大力引进企业总部、科研院所，集聚高端创新要素和生产要素。三是优化综合交通网络。加快城际铁路、客运枢纽、国省道、县乡干线公路建设，推进轨道交通、快速路网、跨江通道、主次干道和公共交通建设。四是统筹城市化发展。推进新型城市化、城乡一体化、全域都市化，加快构建“一核两翼多节点”网络化大都市格局。

　　到2016年，城市现代化基础设施更加完善，国际化品位明显提高，中心城区集聚辐射能力进一步增强，形成产城联动、现代都市融合发展格局。城市化率超过71%，科技研发、国际贸易、港航服务、商务会展、文化创意等功能和总部经济集聚水平大大提升，城乡交通网络体系更加完善，城乡发展规划、产业布局、基础设施、资源配置等基本实现一体化。

　　（四）大力实施民生福祉改善工程。一是积极打造“美丽宁波”。以国家低碳城市试点、“三治理一提高”、“四边三化”、“双清”行动为抓手，着力建设一批生态环境保护设施，加强生态保护与治理，深入实施森林系列创建活动，推进绿色、低碳、可持续发展。二是不断优化人居环境。加强城市景观绿化建设、交通拥堵整治、主次干道和背街小巷整治、城中村和老旧住宅小区改造，实施新一轮打通“断头路”行动，推进农村生态环境整治，加快幸福美丽新家园建设。三是加快社会事业繁荣发展。推进文化教育、医疗卫生、社会保障、养老服务、住房保障等社会事业和民生领域建设。

　　到2016年，基本建成国家级生态市，城乡靓化、绿化、洁化水平大大提升。环境空气质量优于国家环保模范城市标准，新增公共绿地560万平方米，社会事业均衡发展水平和服务能力明显提高，社会保障全民覆盖，住房保障体系基本形成，总体实现基本公共服务均等化。

　　（五）大力实施发展基础强化工程。一是增强海港、空港服务能力。完善“三位一体”港航物流服务体系和海铁联运基础设施，加快机场三期等工程建设。二是增强要素资源供给能力。大力发展清洁能源，科学开发利用滩涂资源，积极拓展建设用地空间，推进地下空间开发，加大人才引进载体建设和人才培养力度。三是深化智慧城市建设。完善智慧城市基础设施，推进智慧交通、智慧健康等应用项目建设。

　　到2016年，港口和机场服务功能更加完善，城市发展的要素资源支撑和信息化服务能力进一步增强。港口吞吐能力不断提高，机场客货枢纽体系更加完善，无线宽带网络覆盖率超过98%，培养高技能人才10万人，人才总量达到170万人。

　　各位代表！为实施好经济社会转型发展三年行动计划，我们将按照市委十二届六次全会的部署，以改革统领各项工作，奋力攻克体制机制的顽疾，突破利益固化的藩篱，摆脱传统路径的依赖，充分发挥市场在资源配置中的决定性作用，让一切可以利用的资源发挥效用，让一切可以激发的活力竞相迸发，力争在民营经济、城乡统筹、对外开放、海洋经济、社会治理、生态环保等重点领域和关键环节改革上取得突破性进展，再创体制机制新优势，切实保障“五项工程”顺利推进，并取得预期成效。

　　三、2014年工作的总体要求、主要目标和重点任务

　　2014年是推进“十二五”规划纲要实施的关键一年，也是全面深化改革的起始之年。今年政府工作的总体要求是：全面贯彻党的十八大和十八届二中、三中全会精神，认真落实省委、省政府“五水共治、五措并举”的要求，把改革创新贯穿于经济社会发展各个领域各个环节，深入实施“六个加快”战略，按照“双驱动四治理”决策部署，扎实启动经济社会转型发展三年行动计划，稳中求进、改中求活、转中求好，强化创新驱动发展，强化城市经济培育，强化生态文明建设，推进城乡一体化，推进城市国际化，推进基本公共服务均等化，努力保持经济持续健康发展、社会和谐稳定，为实现“两个基本”、建设“四好示范区”奠定更加坚实的基础。

　　综合考虑发展趋势和工作导向，建议今年经济社会发展的主要预期目标为：地区生产总值增长8.5%，地方财政收入增长8%，市区居民人均可支配收入、农村居民人均纯收入分别增长10%和11%；城镇新增就业12.8万人，城镇登记失业率控制在3.6%以内，居民消费价格指数控制在103.5%左右，节能减排指标完成国家和省下达的任务。

　　围绕上述目标要求，今年重点做好六个方面的工作：

　　（一）加快城市经济发展，构筑城乡一体化发展新格局

　　围绕增强城市经济发展基础支撑，推进城市总体规划修改和报批，开展土地利用总体规划修改工作，完善中心城区统筹发展机制，着手行政区划优化布局研究。大力开展“五水共治”，完善三年行动方案，推进水气共治、城乡共治。加强水利基础设施、排水防涝设施建设，加快甬江流域治理，做好姚江分洪排涝工程前期工作。优化市域基础设施布局，推进供水供电、治污纳污、地下管网设施共建共享。加快甬金铁路、港口支线和宁波至奉化、至余慈城际铁路前期工作，开工建设机场三期、三门湾大桥及接线工程，继续推进南北环快速路、新江桥等项目，确保轨道交通1号线一期、铁路货运北站建成投用。

　　强化区域统筹，增强城市发展能级。提升中心城区建设国际化品位，推进重点功能区块开发，加快“两心一轴、三江六岸”城市核心景观带建设，加强历史文化街区保护和背街小巷整治。注重城市精细化管理，启动园林绿化、内河水质、景观照明“三大提升工程”，推进智慧城管二期建设。深入开展交通拥堵整治，推进公交都市建设。加强余慈地区基础设施共建共享、重点产业共兴共转。加大象山港区域保护和利用，有序推进宁波三门湾区域开发建设。提升副中心城市集聚发展水平，加快卫星城市试点镇、中心镇改革发展。

　　培育壮大城市经济主导产业。推进服务业综合改革试点，做好国家级现代服务业示范区、国家示范物流园区申报工作，推进服务业产业集聚示范区和产业基地建设。加快东部新城门户区、国际贸易展览中心拓展区、空港国际物流园区和宁南贸易物流区建设。扶持现代物流、商务中介、文化创意、服务外包等产业，强化旅游业特色发展，推进宁波广告产业园区建设。完善金融产业体系，做大做强地方金融法人机构，发展新型金融业态，加快国际金融服务中心二期建设。积极创建国家电子商务示范城市，启动电子商务“一城两区一中心”建设。完善总部经济政策体系，支持本土总部企业发展。强化区域间专业化分工协作，促进产业优势互补、错位发展，推进县域经济向城市经济转型。

　　以更大力度推进以城带乡、城乡一体。编制新型城镇化规划，稳步推动农业转移人口市民化，统筹城乡基础设施、公共服务设施和社区建设，促进城乡公共资源协调配置。加强区域村庄布局规划，打造城镇都市型新村、田园宜居型靓村、生态文化型美村，建设让人们留恋不舍、记得住乡愁的幸福美丽新家园。研究建立城乡统一的建设用地市场，探索农村宅基地制度改革，推进农村集体经营性建设用地流转试点，提高征地补偿标准。切实帮扶相对欠发达地区加快发展。

　　（二）拓展内外需求，开创经济稳定增长新局面

　　促进投资稳定增长和结构优化。加大城市经济、基础设施、节能环保、“三农”等领域投资。加快重大项目、重点功能区块开发的前期工作，强化要素保障，推进浙江液化天然气接收站二期、上海大众汽车整车等项目。完善民间投资政策，推动民间资本进入基础设施、基础产业、金融服务、文化教育、医疗卫生、健康养老等领域。强化招商选资，实际利用外资34亿美元，引进内资570亿元，浙商甬商回归投资项目实到资金完成省定任务。固定资产投资增长12%。

　　完善促进消费政策措施，进一步扩大信息消费、养老消费、健康消费，推动智能家电、节能汽车、新型电子产品等热点商品消费，鼓励新兴消费方式发展。拓展生活服务消费，完善家政服务体系，加快餐饮住宿业转型发展。统筹商贸综合体、城市商业广场和乡镇商贸中心建设，培育夜市经济街区，加快商品交易市场提升发展，增强“菜篮子”、“米袋子”的保障能力。合理调整最低工资标准，提高低收入群体的收入。办好食博会、宁波购物节等大型商贸展会。社会消费品零售总额增长12%。

　　扶持企业开拓国内外市场，积极推进电子商务拓市场工程，建立网上宁波名品商城，支持宁波产品进入国内外零售企业销售和采购系统，促进市内市外市场融合互补、网上网下市场联动对接。支持企业巩固传统出口市场，鼓励企业开拓内外新兴市场。深化“外贸实力效益工程”和“外贸育苗工程”。加强国际贸易摩擦应对。外贸自营出口增长5%以上，进口增长4%。

　　（三）强化创新驱动，激发转型发展新动力

　　深入实施“科技领航计划”和科技服务专项行动，加强产学研协同创新，推进科技与经济紧密结合、信息化与工业化深度融合，研究与试验发展经费支出占地区生产总值比重提高到2.35%。加快新材料科技城、国家高新区“一区多园”建设步伐，充分发挥“千人计划”余姚产业园、大学科技园、研发园区、和丰创意广场在创新引领、产业带动等方面的作用。支持创新要素向企业集聚，鼓励发展企业工程（技术）中心、科技企业孵化器、公共技术服务平台、产业技术创新战略联盟和企业职工创新团队，开工建设吉利—沃尔沃中国设计中心和试验中心。加强知识产权运用和保护，积极争取国家知识产权局专利审查协作浙江（宁波）中心落地，发明专利授权量达到2600件。完善科技金融服务体系，培育科技中介服务机构、技术交易市场，推动科技成果落地转化。以国际视野引进一流的科研机构和高层次创新型人才、经营管理人才。

　　扎实推进战略性新兴产业发展，加强“810实力工程”企业和高成长企业培育，建设战略性新兴产业专业园，力争战略性新兴产业产值增速快于规上工业产值增速。深化“四换三名三创”工程，推动传统产业改造提升，加快淘汰落后产能。积极支持企业向研发设计、品牌运营和高端制造方向发展，鼓励龙头企业创一流，扶持创新型中小微企业向“专精特新”方向发展。开展工业企业和工业园区单位资源产出绩效评价，将评价结果与要素供应、财政扶持政策挂钩。

　　构建现代农业经营体系。加快现代农业先导区建设，新开工现代农业园区40个，新增标准化粮食生产功能区10万亩。健全农业社会化服务体系，培育家庭农场、股份合作和专业合作组织等新型农业经营主体。推进农业科技创新，扶持现代种业、生态循环农业发展，深化新型业态农业试点。加大农产品质量安全监管和检测力度。加强农田水利基础设施建设和管护，提高农业机械化装备水平。大力培养新型农民。加大强农惠农富农政策力度，强化农村集体资金、资产、资源规范化管理和农民负担监管。发展林下经济。

　　推进海洋经济创新发展示范工作，大力发展海洋高技术产业，培育海洋经济发展新增长极。深化“三位一体”港航物流服务体系建设，提升大宗商品交易所、航运交易所建设水平，打造甬易第三方支付平台，推动东海航运保险公司筹建。加快港口综合开发和海铁联运发展，推进国际卫生港创建。港口货物和集装箱吞吐量均增长5%。

　　把智慧城市建设与产业转型发展、城市功能提升紧密结合起来。完善信息基础设施，深化“三网融合”和“两岸无线城市”试点，完成光网城市三年行动计划。推进智慧健康、智慧物流、智慧交通、智慧教育等试点。开展国家信息消费试点城市和信息惠民工程示范城市建设。加快电子政务大数据布局。推动杭州湾新区和国家高新区智慧产业基地建设，集聚一批物联网、云计算与云服务等智慧产业。

　　深化质量强市建设。健全宏观质量工作体系，完善政府质量绩效考核评价机制。扎实开展质量安全、质量提升、质量创新和质量惠民工程，突出抓好产品、工程、服务和环境质量建设，总体质量保持国内领先水平。

　　（四）推进城市国际化发展，争创开放合作新优势

　　全面实施城市国际化行动纲要，推动新一轮对外开放，提升开放型经济发展水平。积极打造国际贸易中心城市，推进市场采购监管模式、跨境贸易电子商务服务和进口贸易便利化改革试点，深化国家进口贸易促进创新示范区建设，推动国际贸易转型升级。大力发展进口贸易，提高进口商品展示交易中心、梅山保税港区汽车整车进口口岸的建设运营水平。创新招商引资机制，推进引资、引智、引技相结合。鼓励市场化办展办会，支持行业协会举办贸易展会。积极承办中东欧国家经贸促进部长级会议及商品展，精心筹备2014年亚太经合组织首次高官会议，办好浙洽会、消博会等重点展会。加强和改进口岸工作。积极培育本土跨国企业，支持企业开展跨国并购，提升境外基地的规模水平，完成境外中方投资额18亿美元，境外承包工程劳务合作营业额增长12%。

　　构建开放平台新优势，加快宁波综合保税区“一区多片”申报，整合优化海关特殊监管区域，推进宁波象保合作区建设。推动大榭开发区与县（市）区产业合作，探索境内外合作开发园区新途径。促进重点开发区域错位发展，做强主导产业，大力引进技术含量高、带动能力强、产业链条长的好项目大项目。宁波杭州湾、梅山产业集聚区各引进1个以上50亿元产业大项目。

　　深化国际国内交流合作。办好“宁波周”活动，推进与国内外重点区域、重要城市的合作交流，全面扩大和提升甬台经贸合作交流。推动浙东经济合作区一体化发展，增进与长三角城市的合作。主动对接上海自由贸易试验区建设，加快宁波—舟山港一体化进程，推进“无水港”、港口联盟建设。加强与“宁波帮”和帮宁波人士的联络。继续推进对口援助和山海协作。

　　（五）保障和改善民生，不断回应人民群众新期盼

　　建设文化强市，努力让人们置身城市之中，处处能够感受到高尚的城市文明、较高的文化品位和浓郁的文化气息。推进文明城市创建常态化、长效化，争创全国文明城市“四连冠”。培育和践行社会主义核心价值观，深化志愿服务活动，推进公民道德建设。开展文化惠民活动，完善群众评价和绩效评估机制，促进公共文化服务标准化、均等化、社会化发展。加强文化遗产管理，推进大运河（宁波段）申遗，加快海洋渔文化生态保护区建设，中国港口博物馆和国家水下文化遗产保护宁波基地落成开放。深入实施文化发展“1235”工程，培育壮大传媒产业，支持中小文化企业发展，推进钓鱼台·美高梅“中国假期”、华强·中华复兴文化园、华侨城文化旅游等项目。进一步扩大对外文化交流。

　　深化教育改革发展。发展普惠性民办幼儿园，确保60%以上幼儿入读普惠性幼儿园。统筹城乡义务教育发展，所有县（市）区通过全国义务教育发展基本均衡县（市）区验收。推进普通高中多样化、特色化发展，启动东部新城高中建设。实施高校重点学科和专业提升计划，大力发展职业教育，深化产教融合、校企合作，引进中英时尚学院、中德汽车学院等二级学院，启动国家级职业教育与产业协同创新试验区建设，加强高素质劳动者和技能型人才培养。加快终身学习公共服务平台、数字化学习平台建设，完善全民终身教育体系。重视青少年体质健康教育，推进社会实践大课堂建设。健全校园安全“三防”联防体系，推进百万学生餐饮安全工程，保障学生食品安全和上下学交通安全。

　　提高就业和社会保障水平。实施更加积极的就业政策，深化国家级创业型城市和充分就业县（市）区建设，完善城乡均等的公共就业创业服务体系，加大对高校毕业生、农村转移劳动力、城乡就业困难人员、退役军人等帮扶力度。统筹城乡一体化社保体系建设，推进被征地人员养老保障制度与基本养老保险制度衔接转换，实现城乡居民医保管理职能和经办服务的整合，实施城乡居民大病保险办法，本地户籍人口养老保障参保率提高到88.5%。深化社会养老服务体系改革，健全社会救助制度，完善残疾人社会保障和服务体系。

　　完善住房保障制度，进一步改善困难群众的住房条件，实现不同保障方式的合理衔接。新开工保障性安居工程209万平方米、2.7万套，完善保障性住房的周边公共配套和后续管理服务。深化“三改一拆”行动，加快城中村、老旧小区、非成套房、危房改造和农村住房集中改建。推进房地产市场健康发展。

　　加快公立医院综合改革，开展按病种收费方式改革试点，完善基本药物制度和基层医疗卫生机构运行机制。探索合理的分级诊疗模式，建立社区医生和居民契约服务关系。加快新十大医疗卫生基础设施建设，启动市第一医院西部院区项目，开工建设宁波杭州湾医院。加强与境内外知名医疗机构、医学院校合作，推进李惠利医院东部院区与台北医学大学合作办医，加快浙大宁波医院建设。加强公共卫生和爱国卫生工作。积极创建体育强市，启动奥体中心项目。积极稳妥实施“单独两孩”生育新政策，促进人口长期均衡发展。

　　构建科学有效的社会治理体制。促进行业协会、公益慈善等社会组织发展，推动“网格化管理、社会化服务”工作体系与基层和谐促进网络紧密衔接。加大社会矛盾预防、排查和调处，推进领导干部接访下访常态化。

　　加强基层综治组织建设，完善立体化社会治安防控体系。坚持正确的舆论导向，完善新闻发布体系，推动传统媒体与新兴媒体融合发展，加强网络管理，规范传播秩序。进一步创新流动人口管理服务，优化流动人口结构。加强民族、宗教工作。严格落实安全生产责任制，推进地下油气管线安全隐患排查治理，强化建设施工和高危领域的安全监管。加强海洋防灾减灾体系建设。深化食品药品监管体制改革，开展食品安全责任保险试点，推进小餐饮规范整治。积极构建和谐医患关系，妥善处理劳资纠纷。强化应急管理，提高突发事件应对能力，开展巨灾保险制度试点。加强国家安全工作。大力支持驻甬人民解放军和武警部队建设，推进国防后备力量和人民防空建设，创新双拥工作机制，推动军民融合深度发展。

　　各位代表!为切实保障和改善民生，市政府今年将继续办好一批民生实事，让人民群众更好地共享改革发展成果。

　　（六）加强生态文明建设，展现“美丽宁波”新面貌

　　“铁腕”推进“三治理一提高”行动。尽最大努力加强雾霾治理，开展空气质量指数预报，健全大气重污染预警和响应机制。控制煤炭消费，提高天然气等清洁能源使用率，扩大“禁燃区”范围，加快淘汰黄标车。加大重污染行业治理，开展重点区域污染整治，加强建设工程、码头作业场所扬尘和餐饮油烟污染整治，坚决制止垃圾、秸秆露天焚烧行为。重视抓好饮用水源地环境保护，强化城区内河和农村河网水环境综合整治，推动中水利用，加强农业面源污染和近海海洋环境污染治理。强化重金属污染综合防治和废塑料加工行业整治，推进生活垃圾无害化处理，完善固体废物污染防治设施体系，积极开展土壤污染的治理修复。

　　推进生态环保设施和工程建设。全面完成电力企业脱硝工程，加快城镇污水污泥处理设施建设，推进鄞西污水处理厂一期、北区污水处理厂二期等项目，建成运行一批危险废物处置设施，加强治污减排设施运行监管。深化国家循环经济示范城市建设，实施工业循环经济“611”工程、清洁生产企业示范工程，推进开发园区生态化、循环化改造，大力发展节能环保产业。加快国家低碳城市试点建设。深化“四边三化”、“双清”等行动，加强山区水源涵养林建设，提高平原地区绿化美化水平。

　　创新生态环境保护体制机制。开展新一轮生态文明建设规划和生态功能区划修编，划定生态保护红线，完善生态绿地空间规划。实行差别化区域开发管理政策，完善重点生态功能乡镇绩效考核评价办法。强化能耗强度和能耗总量“双控”节能目标责任制。深入实施排污权有偿使用和交易制度，全面推行排污许可证制度。加大对生态功能重点保护区域的财政补贴和转移支付力度，提高生态补偿标准。加强污染联防联控管理，全面实行“河长制”。加强环境保护制度和执法力量建设，对一切环境违法行为实行“零容忍”，毫不留情坚决予以严惩。

　　四、推进改革和政府自身建设

　　今年经济社会发展任务十分繁重，必须以改革为突破口。市政府将认真贯彻落实中央和省委、市委关于全面深化改革的决定要求，正确把握，勇于进取，稳扎稳打，统筹推进，以更大的胆魄、更快的步伐、更实的举措，抓紧出台各项改革具体实施方案。对方向明确、政策清楚的改革，不等不看，不失时机，马上改、改到位，争取走在前列；对面上推动难度较大、上级纳入试点的改革，积极探索，敢为人先，力争取得突破性进展；对需要上级决策的改革，不盲动、不抢跑，认真细致做好准备工作，适时、稳妥启动改革。

　　加强政府自身建设是做好改革和发展工作的切入点。市政府将按照“为民、务实、清廉”的要求，正确处理政府与市场、政府与社会的关系，以政府职能转变和服务管理的改革创新，推动各项改革举措落到实处，促进经济社会全面发展。

　　（一）优化审批，简政放权。最大限度减少行政审批事项，继续取消和下放一批审批权限，优化审批环节，缩短审批时限，深入推进行政审批标准化、便利化建设，探索对负面清单外符合市场准入标准的项目实行零审批。对不符合深化改革要求的行政权力依法予以严管、暂停或取消。深化以职能转变、精简机构、精简人员为重点的政府机构改革，优化机构设置和职能配置。推进政事、政社分开，放开应该由企业和社会组织自我服务、自我管理的事项，推行政府购买服务。

　　（二）改进管理，强化服务。加强发展战略、规划、政策的制定、实施和督查，启动“十三五”规划前期研究。强化政府目标管理，完善绩效管理考评体系。盘活财政资金存量，推进部门预算公开，加大对社会事业和民生领域的支出，增加对相对欠发达地区的转移支付。加快地方金融改革，系统创建金融生态示范区，扩大非传统信贷融资，鼓励企业有效利用多层次资本市场，扩大直接融资规模。完善建设资金和政府债务统一管理制度，合理控制政府债务。健全经营性国资监管体制，整合重组国有企业资产，发展混合所有制经济，完善国企现代企业制度。坚决打破民间投资隐性壁垒，推进工商登记制度改革，完善社会征信体系，增强政策咨询服务平台功能。加大市场价格监管力度。加强基层防灾减灾体系建设，提高重大气象灾害监测预报预警水平。

　　（三）转变作风，提高效能。健全改进作风长效机制，完善调查研究和联系服务群众制度，进一步改进会风文风。完善政务公开和各领域办事公开制度，推进决策、管理、服务、结果“四公开”。推进政务热线整合，加快信访制度改革，畅通群众诉求渠道。强化行政效能监督和问责。全面推行干部绩效对账制度，加强公务员培训、监督和管理，倡勤治懒，建设一支勤政高效的公务员队伍。

　　（四）依法行政，勤政廉洁。规范行政行为，严格按法定权限和程序履职，加快法治政府建设。自觉接受人大法律监督、工作监督和政协民主监督，主动听取民主党派、人民团体和各界人士的意见建议，重视舆论监督、公众监督，让政府工作置于全方位监督之下。强化权力公开规范运行，健全和落实重大决策、行政审批、权力清单公开制度和常态化工作机制，推进公共资源交易平台整合，突出对行政审批、工程招投标、土地转让、政府采购等重点领域的监管和审计监督。深化行政执法体制改革，推进严格规范公正文明执法。强化源头治理、制度防腐，保持惩治腐败高压态势。大兴艰苦奋斗之风，厉行勤俭节约、反对铺张浪费，严格控制一般性支出，确保“三公”经费继续下降。

　　各位代表！全面完成今年各项目标任务，关键在于实干。让我们更加紧密地团结在以习近平同志为总书记的党中央周围，深入贯彻落实党的十八大和十八届二中、三中全会精神，在中共宁波市委的领导下，团结奋进，开拓创新，鼓足劲，扎实干，出实效，为实现“两个基本”、建设“四好示范区”而努力奋斗！
